# Color Image Generation from Range and Reflection Data of LiDAR

**DOI:** 10.3390/s20185414

**Published:** 2020-09-21

**Authors:** Hyun-Koo Kim, Kook-Yeol Yoo, Ho-Youl Jung

**Affiliations:** Department of Information and Communication Engineering, Yeungnam University, Gyeongsan 38544, Korea; kim-hk@ynu.ac.kr (H.-K.K.); kyoo@yu.ac.kr (K.-Y.Y.)

**Keywords:** artificial intelligence, fusion technique, heterogeneous source, image generation, LiDAR range, LiDAR reflection, sparse input

## Abstract

Recently, it has been reported that a camera-captured-like color image can be generated from the reflection data of 3D light detection and ranging (LiDAR). In this paper, we present that the color image can also be generated from the range data of LiDAR. We propose deep learning networks that generate color images by fusing reflection and range data from LiDAR point clouds. In the proposed networks, the two datasets are fused in three ways—early, mid, and last fusion techniques. The baseline network is the encoder-decoder structured fully convolution network (ED-FCN). The image generation performances were evaluated according to source types, including reflection data-only, range data-only, and fusion of the two datasets. The well-known KITTI evaluation data were used for training and verification. The simulation results showed that the proposed last fusion method yields improvements of 0.53 dB, 0.49 dB, and 0.02 in gray-scale peak signal-to-noise ratio (PSNR), color-scale PSNR, and structural similarity index measure (SSIM), respectively, over the conventional reflection-based ED-FCN. Besides, the last fusion method can be applied to real-time applications with an average processing time of 13.56 ms per frame. The methodology presented in this paper would be a powerful tool for generating data from two or more heterogeneous sources.

## 1. Introduction

Light detection and ranging (LiDAR) sensors are widely used for the advanced driver-assistance systems (ADAS) and autonomous vehicles. LiDAR sensors provide information that consists of range (distance) and reflection. LiDAR range data have been used for various applications, such as semantic segmentation [1,2,3,4], 3D mapping [5,6], and object detection [7,8,9,10,11,12,13,14]. On the other hand, reflection data have been utilized for the recognition of driving-related factors in the environment, such as lanes, road marks, and traffic signs, which have relatively high reflectivity [15,16,17,18].

**Figure 1:**
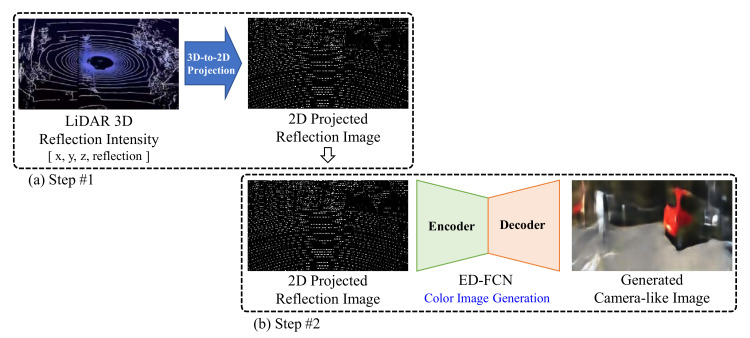
The camera-captured-like color-image-generation method using 3D LiDAR point clouds. The method consists of two steps: (**a**) LiDAR 3D-to-2D projection and (**b**) color image generation.

Interestingly, it has been reported that the deep learning-based encoder-decoder structured fully convolution network (ED-FCN) can successfully generate camera-captured-like color images from heterogeneous LiDAR reflection data [19,20,21]. Note that the ED-FCN network is originally applied for semantic segmentation. The methods [19,20,21] consist of two steps, as shown in Figure 1. In the first step, LiDAR 3D reflection data are projected into 2D color image coordinate. The color image is generated from the projected reflection data in the second step.

In [19], a low-complexity ED-FCN network was used for camera-captured-like gray-scale image generation. Note that the monochrome images were generated from the LiDAR reflection data. The projected reflection image had different sparsity and characteristics, compared with the target image. To increase better generation performance and generate color image, an asymmetric ED-FCN, i.e., a decoder, with greater depth than the encoder, was proposed in [20]. The asymmetric ED-FCN outperformed symmetric ED-FCN and the generative adversarial network (GAN)-based colorization method [22]. Recently, a selected connection UNET (SC-UNET) was proposed in [21], which considers the sparseness of each level in the encoder network and the similarity between the same levels of encoder and decoder networks. The SC-UNET with the connection between encoder and decoder at the two lowest levels outperforms the SC-UNET with other connections and the asymmetric ED-FCN. One interesting result discussed in [19,20,21] was that shadow-free images were generated since the LiDAR reflection data were originally produced, irrespective of the illumination. Note that these methods [19,20,21] generate an image only from the reflection data of LiDAR.

In this study, we firstly tried to generate color images using the LiDAR range data. The simulation result confirms that the color image can be also generated from the range data. Accordingly, we propose three color-image-generation methods which fuse both reflection and range data to improve the quality of generated image—early fusion, mid fusion, and last fusion-based LiDAR to color-image-generation methods. KITTI-based evaluation dataset was used for training and performance verification [20,21]. Peak signal-to-noise ratio (PSNR) [23] and structural similarity index measure (SSIM) [23,24] metrics were used for the performance evaluation. The number of weights and the average operation time of the network were used for the comparison of the computational complexities of various image generation networks. The proposed fusion methods improve image generation performance over the conventional image generation methods which use only reflection data. In addition, the proposed fusion methods have operating times applicable to real-time applications. The simulation results show that the proposed fusion methods give better performance compared reflection-only methods.

The rest of this paper is organized as follows. In Section 2, we propose fusion network architectures which generates a camera-captured-like 2D color image from both the range and reflection 3D LiDAR data. The training and inference processes are also described. In Section 3, the performances of the proposed networks are compared with the performance of the conventional reflection-based ED-FCN network. Additionally, the range-based ED-FCN network is compared. Section 4 draws the conclusions.

## 2. Proposed Method

The conventional color-image-generation methods [19,20,21] use only LiDAR reflection data. To verify the possibility of color image generation from range data, the range data are applied to the conventional image generation networks. To improve the color image generation performance, we propose three-color image generation networks using LiDAR range data and reflection data. Considering that LiDAR range and reflection data have different characteristics, fusion network architectures were proposed and evaluated through experiments.

### 2.1. Transformation of 3D LiDAR Point Cloud

The 3D LiDAR point cloud ${\left[X,Y,Z,R\right]}^{T}$ provided by the LiDAR sensor consists of the world coordinate position ${\left[X,Y,Z\right]}^{T}$ and the reflection data R(X,Y,Z) of the object. The 3D LiDAR point cloud is projected onto the coordinate ${\left[u,v\right]}^{T}$ of the color image to be generated by using Equation (1).

(1) $$s\left[\begin{array}{c} u\\ v\\ 1 \end{array}\right]=\left(\begin{array}{ccc} f_{u} & 0 & c_{u}\\ 0 & f_{v} & c_{v}\\ 0 & 0 & 1 \end{array}\right)\left(\begin{array}{cccc} 1 & 0 & 0 & 0\\ 0 & 1 & 0 & 0\\ 0 & 0 & 1 & 0 \end{array}\right)\left(\begin{array}{cc} R_{L}^{C} & t_{L}^{C}\\ 0 & 1 \end{array}\right)\left[\begin{array}{c} X\\ Y\\ Z\\ 1 \end{array}\right]$$

where *s* indicates the scale factor, $f_{k}$; $c_{k}$ and k=u,v are focal length and principal point of the camera, respectively; and $R_{L}^{C}\in R^{3\times 3}$ and $t_{L}^{C}\in R^{1\times 3}$ represent the rotation and translation matrices for LiDAR-to-camera transform, respectively.

The 2D reflection images are obtained according to Equation (2). Practically, several 3D LiDAR points, $(X_{i},Y_{i},Z_{i}),i=1,\cdots ,N$ provided by a LiDAR sensor may be projected onto the same location of (u,v) in the image plane. In this case, the reflection value, r(u,v) is determined by the average of the reflection values of the *N* LiDAR points.

(2) $$r\left(u,v\right)=\frac{1}{N}\sum_{i=1}^{N}R\left(X_{i},Y_{i},Z_{i}\right)$$

The 2D range images are calculated by using Equation (3). Similarly, for *N* 3D LiDAR points projected onto the same location of (u,v), the distance value, d(u,v), is determined by the minimum of the distance values of the *N* LiDAR points.

(3) $$d\left(u,v\right)=\min_{i=1\ldots N}\sqrt{X_{i}^{2}+Y_{i}^{2}+Z_{i}^{2}}$$

### 2.2. Single-Input-Based Color Image Generation

**Figure 2:**
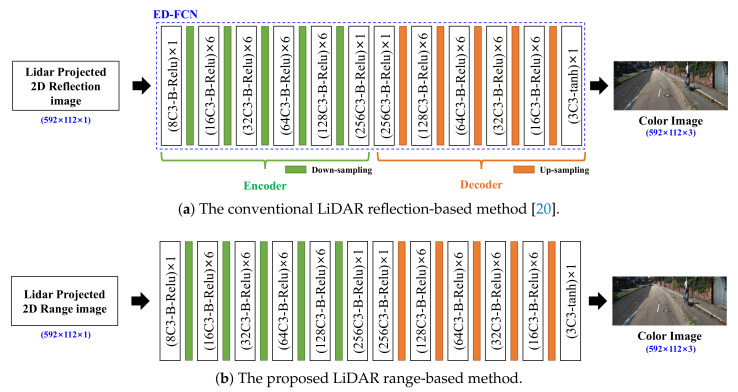
The color camera-captured-like image generation methods using 2D projected (**a**) reflection and (**b**) range data.

The color-image-generation methods from the LiDAR 2D projected reflection or range image are shown in Figure 2.

#### 2.2.1. LiDAR Reflection-Based Method

Figure 2a shows the ED-FCN-based color-image-generation method by using only a reflection image proposed in previous works [19,20]. The ED-FCN network generates the RGB color image (size: 592 × 112 × 3) from the sparse 2D reflection image (size: 592 × 112 × 1). In Figure 2, each block in the network is expressed by ($N_{f}$C$N_{k}$-B-$f_{a}$)$\times N_{b}$ where the $N_{b}$ is the number of convolution blocks and each convolution block is composed of the convolution layer with $N_{f}$ filters with ($N_{k}\times N_{k}$) kernel size, a batch-normalization [25] layer, and $f_{a}$ kind of activation function. For example, (16C3-B-Relu) × 6 means six of the convolution blocks, each of which includes the convolution layer of 16 filters with (3 × 3) kernel size, the batch-normalize layer, and the ReLU [26] activation function which is repeated six times. As another example, (3C3-tanh) × 1 means one convolution block including the convolution layer with 3 filters of (3 × 3) size and the tanh [27] activation function. In each convolution layer, stride 1 and zero-padding are commonly applied. For down and up samplings, max-pooling and un-pooling [28] with a factor of 2 are applied, respectively.

#### 2.2.2. LiDAR Range-Based Method

Figure 2b shows a method which uses the same network of Figure 2a but input is replaced with the range image instead of the reflection image to verify the image generation performance according to the type of LiDAR data.

### 2.3. Proposed Multi-Input-Based Color Image Generation

In applications with two or more multi-input datasets with different characteristics, fusion networks are classified into three types: early fusion [1,2,9,15], mid fusion [3,7,8,9,10,11,29], and last fusion [2,13] methods, depending on where the data are combined together. In the case of the early fusion method, multiple sources are concatenated into a single input before being applied to the image generation network. The mid fusion method merges the features of the intermediate output of each network with different input data. The last fusion method combines the outputs of each network into a final output.

**Figure 3:**
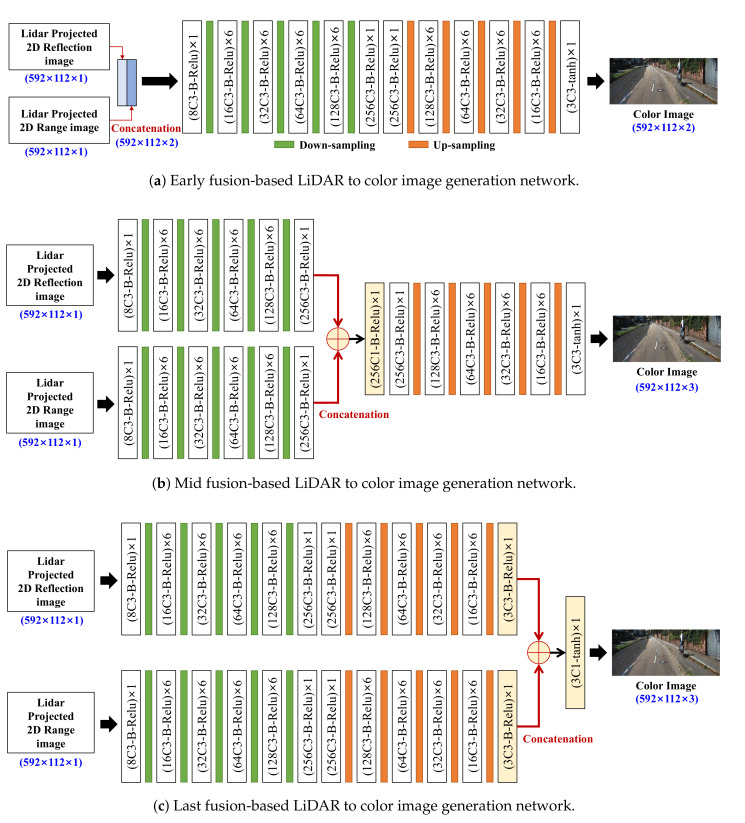
The proposed fusion-based LiDAR to color image generation networks using LiDAR reflection and range data: (**a**) early fusion, (**b**) mid fusion, and (**c**) last fusion-based LiDAR to color image generation networks.

Figure 3 shows the proposed fusion-based LiDAR to color image generation networks using two heterogeneous datasets, i.e., LiDAR reflection and range data, where ED-FCN is used.

#### 2.3.1. Early Fusion-Based LiDAR to Color Image Generation Network

The projected 2D reflection and range images are concatenated in channel direction to form an input. For instance, reflection and range images with the resolution of (592 × 112 × 1) are combined into a (592 × 112 × 2) concatenated image. The concatenated input is applied to the same network as shown in Figure 2. The overall architecture of the early fusion network is depicted in Figure 3a.

#### 2.3.2. Mid Fusion-Based LiDAR to Color Image Generation Network

For the mid fusion network, as shown in Figure 3b, two encoder networks independently extract the feature-maps from the projected 2D range and reflection images, respectively. The two feature-maps are merged by concatenation operation. Additionally, then, the convolution layers with the 256 (1 × 1) filters, batch-normalize layer, and ReLU activation are repeatedly applied. Finally, the color image is generated by using a decoder network which is the same decoder network used in image generation network with single input.

#### 2.3.3. Last Fusion-Based LiDAR to Color Image Generation Network

For the last fusion network, as shown in Figure 3c, two decoder feature maps are independently extracted from the projected 2D reflection and range images by using single-input-based image generation network. It should be noted that the activation functions of the last convolution layer use ReLU activation, unlike other networks wherein tanh is used. The resultant feature-maps are also merged by using a concatenation. The final color image is generated by using convolution layer with the three (1 × 1) filters and following tanh activation.

### 2.4. Training and Inference Processes

We used a dataset [19,20] composed of a triplet of sparse LiDAR 2D projected reflection images, sparse 2D projected range images, and their corresponding dense RGB color images. The projected reflectance and range images were obtained from a LiDAR 3D point cloud with reflection intensity through Equations (2) and (3). The color images synchronized with the reflection and range data were captured from the camera and were used for ground truth (GT) data. In the case of the single-input-based image generation network shown in Figure 2, the 2D reflection image or range image was used. Additionally, in the multi-input-based image generation network shown in Figure 3, both the reflection image and the range image were used as input data of the network. The range and reflection data are normalized into [0,1], before feeding to the network.The target image of the image generation network, in other words, GT image, is the RGB color image which corresponds to the two LiDAR 2D projected images. As the tanh activation function [27] in the last layer in the image generation networks is used, the dynamic range of generated output data is confined to [−1,1]. The target color images are, therefore, normalized to have the same dynamic range during the training process. As in the previous works [19,20,21], mean squared error (MSE) is used as a loss function for training.

For training, the proposed network architectures are trained by using adaptive moment estimation (Adam) solver [30] with a maximum of 2000 epochs.All other parameters are the same as those of the Adam solver used in [20]. The early stopping technique with a patience parameter of 25 is applied for validation loss [31].

In the inference process, three-channel output data having the dynamic range [−1,1] are generated and normalized for the final RGB color images to have the dynamic range of [0,255].

## 3. Experimental Results

### 3.1. Simulation Environment

#### 3.1.1. Evaluation Dataset

The evaluation dataset was reconstituted from the KITTI raw dataset [32], as in [19,20]. The dataset consists of a triplet of images—a projected 2D LiDAR reflection image, a 2D LiDAR range image, a and color image—that were recorded simultaneously. The reflection and range images were used for the input and the color image was used for ground truth. The triplets of the images that were recorded under heavy shadows were manually excluded in the evaluation dataset, because one advantage of the proposed method is to generate shadow-free color images from LiDAR data. The evaluation dataset consisted of 4308 triplets—2872 triplets for training, 718 for validation, and 718 for testing as used in [19,20]. All evaluation images had the same resolution of 592 × 112 (66,304 pixels). Both the reflection and range images had on average 3502 valid values. In other words, the density of both the projected 2D LiDAR images was 5.28% [21]. Therefore, both the LiDAR images were very sparse and even irregular compared to the target RGB color image.

#### 3.1.2. Measurement Metrics

To evaluate the image quality between the generated color image and target color image, PSNR [23] and SSIM [23,24] were used. In the case of PSNR performance evaluation, since both the generated image and the GT image were in color, PSNR was calculated based on the RGB color image. In other words, PSNRs were separately calculated for each *R*, *G*, and *B* channel, and their average, denoted as PSNR${}_{c}$ was used for the evaluation. Additionally, the gray-scale PSNR, denoted as PSNR${}_{g}$ was measured using only the gray-scale *Y* component between the generated and the GT images. For the SSIM, only the gray-scale *Y* component was used.

For the evaluation of the complexity, the average inference computation time and the total number of weights of the network were compared. We used a workstation with an Intel Core i7-6850 CPU 3.60 GHz and a Nvidia Titan X Pascal GPU. The software environments were Ubuntu 16.04, Python 3.5.6, Tensorflow 1.13.1 [33], and Keras 2.3.1 [34].

### 3.2. Performances of Single-Input-Based Methods

In this section, the performances of the single-image input-based color-image-generation methods are compared. LiDAR reflection-based and LiDAR range-based networks shown in Figure 2a,b were implemented and evaluated respectively. As mentioned in the previous section, the network was the same, but input data were different.

Table 1 shows the results of the image quality from the single-input-based color image generation network according to the input data type. Average PSNR and average SSIM are listed with standard deviation in parenthesis. The LiDAR range input-based image generation can also generate a color image as the reflection input-based method. However, the range-based network has on average 0.67 dB in PSNR${}_{g}$, 0.64 dB in PSNR${}_{c}$, and 0.05 in SSIM less than the reflection-based network, respectively. The gray-scale PSNR (PSNR${}_{g}$) is higher than color-scale PSNR${}_{c}$, irrespective of input data. The results show the possibility of color image generation from LiDAR range data.

Table 2 shows the computational complexity according to the input data type in the single-input-based color image generation network. As both input-data-based networks use the same image generation network, the number of weights and average inference computation time were the same, 3,350,243 and 6.91 ms, respectively.

### 3.3. Performances of Proposed Multi-Input-Based Methods

For evaluation of the performances of the multi-input-based color-image-generation methods (here, two-inputs: reflection and range), the three fusion networks shown in Figure 3 were tested and evaluated.

Table 3 shows the results of the image quality performances of the multi-input-based color image generation networks. Like the experimental results in Section 3.2, gray-scale PSNR (PSNR${}_{g}$) was higher than the color-scale PSNR (PSNR${}_{c}$), irrespective of fusion type. The results show that the mid fusion-based method had better image generation performance than the early fusion method, and the last fusion was better than the mid fusion. On average, the last fusion method showed higher improvements of 0.35 dB in PSNR${}_{g}$, 0.33 dB in PSNR${}_{c}$, and 0.01 in SSIM over the early fusion method, respectively. Among all image generation methods, including single-input and multi-input-based methods, the last fusion method had the best performance. In addition, on average, the last fusion method achieved a color image generation performance of 20.06 dB in PSNR${}_{g}$, 19.64 dB in PSNR${}_{c}$, and 0.53 in SSIM.

Table 4 shows the computational complexities of the three types of fusion-based networks. The networks used in the early fusion, the mid fusion, and the last fusion-based networks have the about 1.06, 1.45, and 2.00 times of the weights of the single-input-based network, respectively. Additionally, the average computation times of the early fusion, the mid fusion, and the last fusion-based methods are about 1.02, 1.44, and 1.96 times slower than the single-input-based image generation, respectively.

### 3.4. Subjective Image Quality Evaluation

**Figure 4:**
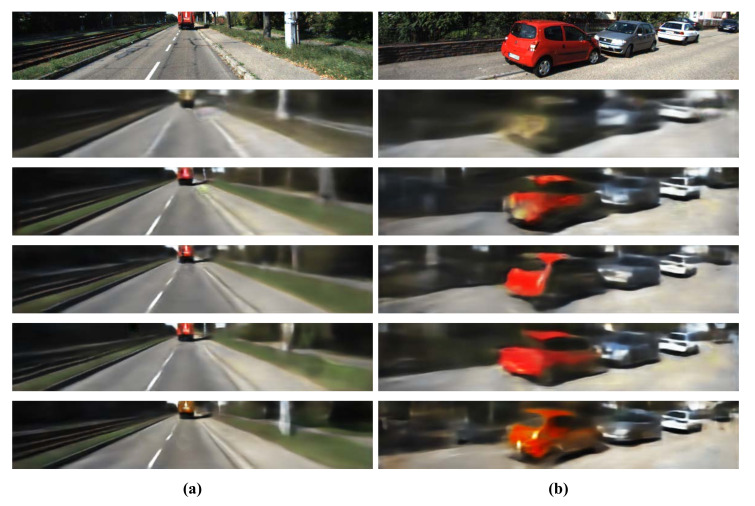
Two representative inference examples. (**a**) shows that the white road-pillar located on the right side does not appear in the reflection-based single-input network. (**b**) shows that the visual qualities are similar to the objective performances shown in Table 1 and Table 3. The GT color image is shown in the top row. The second to sixth rows are the images generated by range-based (in the second row) and reflection-based (in the third row) single-input networks and early fusion (in the fourth row), mid fusion (in the fifth row), and last fusion-based multi-input networks (in the sixth row), respectively.

For the evaluation of subjective image quality, two representative inference examples are given in Figure 4, where GT color images are shown in the first row, and the second to sixth rows are the images generated by range-based (in the second row) and reflection-based (in the third row) single- input networks and early fusion (in the fourth row), mid fusion (in the fifth row), and last fusion-based multi-input networks (in the sixth row), respectively.

All image generation methods generate blurred images compared to GT images. As shown in the second row of Figure 4, the range-based image generation method generated a somewhat camera-captured-like image. However, the range-based method did not produce color information properly and produced the most blurred image compared to the other methods. The images in the third row show that the reflection-based, single-input image generation can generate color images.

The proposed three fusion methods generate color images more faithfully than reflection-based, single-input image generation. That means the LiDAR range data are clearly useful for the color image generation from LiDAR.

One interesting result is the generation of the white road-pillar on the right side of the image shown in Figure 4a; the reflection-based single-input network could not produce the pillar but the range-based network produced it. That means that the LiDAR range data are helpful in generating geometric structures of objects, thereby producing generated images with better subjective qualities. For the cases of the multi-input networks, the mid fusion and the last fusion networks produced the pillar. In particular, the last fusion-based multi-input network demonstrated faithful generation of the pillar.

Figure 4b shows that the five image generation methods have visual performances similar to the objective performance shown in Table 1 and Table 3.

## 4. Conclusions

In this paper, we examined the effectiveness of the LiDAR range data for camera-like color image generation. LiDAR range data are used as input data in the existing reflection-based single-input color generation network that consists of ED-FCN. Through the simulations, we showed that the range-based single-input method can generate camera-like images, even though the visual quality of the generated image is slightly less than for the reflection-based method. Clearly, the LiDAR range data are useful for LiDAR to color image generation. We considered the use of both LiDAR reflection and range data, and then proposed three kinds of fusion networks based on multiple inputs, i.e., two inputs. The early fusion, mid fusion, and last fusion-based LiDAR-to-color image generation networks were designed and tested.

The proposed last fusion-based method achieved higher improvements of 0.53 dB in PSNR${}_{g}$, 0.49 dB in PSNR${}_{c}$, and 0.02 in SSIM over the previous reflection-based single input method. In addition, the last fusion method is applicable to real-time applications with an average processing time of 13.56 ms.

Therefore, these results show that the fusion of the feature-maps of the decoder networks is better than the fusion of the feature-maps of the encoder networks when the input and output of the image generation network have different characteristics. These results can be applied to various applications, such as object recognition, segmentation, and 3D map-generation using LiDAR data and image generation.

## Figures and Tables

**Table 1:** Generated image quality performance using single-input-based color-image-generation methods in terms of average gray-scale PSNR (PSNR${}_{g}$), color PSNR (PSNR${}_{c}$), and SSIM, where standard deviation is listed in parenthesis.

Input Data	Validation			Test		
	PSNR${}_{g}$	PSNR${}_{c}$	SSIM	PSNR${}_{g}$	PSNR${}_{c}$	SSIM
LiDAR reflection	19.57	19.18	0.51	19.53	19.15	0.51
	(±2.49)	(±2.36)	(±0.11)	(±2.47)	(±2.34)	(±0.11)
LiDAR range	18.90	18.55	0.46	18.86	18.51	0.46
	(±2.01)	(±1.95)	(±0.09)	(±2.00)	(±1.94)	(±0.09)

**Table 2:** Complexity performance results of single-input-based color-image-generation methods in terms of the number of weights and average inference processing time.

Input Data	The Number of Weights	Average Processing Time [ms]
LiDAR Refection	3,350,243	6.91
LiDAR Range	3,350,243	6.91

**Table 3:** Image quality performance results of the proposed fusion-based color image generation in terms of average gray-scale PSNR (PSNR${}_{g}$), color PSNR (PSNR${}_{c}$), and SSIM.

Fusion-Based Network	Validation			Test		
	PSNR${}_{g}$	PSNR${}_{c}$	SSIM	PSNR${}_{g}$	PSNR${}_{c}$	SSIM
Early fusion-based network	19.79	19.37	0.52	19.71	19.31	0.52
	(±2.51)	(±2.37)	(±0.11)	(±2.52)	(±2.37)	(±0.11)
Mid fusion-based network	19.96	19.55	0.52	19.91	19.50	0.52
	(±2.46)	(±2.34)	(±0.11)	(±2.44)	(±2.30)	(±0.11)
Last fusion-based network	20.10	19.68	0.53	20.06	19.64	0.53
	(±2.62)	(±2.44)	(±0.11)	(±2.61)	(±2.42)	(±0.11)

**Table 4:** Complexity performance results of the proposed fusion-based color image generation in terms of the number of weights and average inference processing time.

Fusion-Based Network	The Number of Weights	Average Processing Time [ms]
Early fusion-based network	3,550,315	7.03
Mid fusion-based network	4,863,219	9.93
Last fusion-based network	6,700,531	13.56
